# Effect of Vitamin D Supplementation in Early Life on Children’s Growth and Body Composition: A Systematic Review and Meta-Analysis of Randomized Controlled Trials

**DOI:** 10.3390/nu13020524

**Published:** 2021-02-05

**Authors:** Kristine Ma, Shu Qin Wei, Wei Guang Bi, Hope A. Weiler, Shi Wu Wen

**Affiliations:** 1 CHU Sainte-Justine Research Center, Department of Obstetrics and Gynecology, Faculty of Medicine, University of Montreal, Montreal, QC H3T 1C5, Canada; kristine.ma@umontreal.ca (K.M.); wei.guang.bi@umontreal.ca (W.G.B.); 2 Institut National de Santé Publique du Québec, Montreal, QC H2P 1E2, Canada; 3 School of Human Nutrition, McGill University, Montreal, QC H9X 3L9, Canada; hope.weiler@mcgill.ca; 4 OMNI Research Group, Ottawa Hospital Research Institute, Ottawa, ON K1H 8L6, Canada; 5 Department of Obstetrics, Gynecology, and Newborn Care, University of Ottawa, Ottawa, ON K1N 6N5, Canada; 6 School of Epidemiology and Public Health, University of Ottawa, Ottawa, ON K1G 5Z3, Canada

**Keywords:** Vitamin D, pregnancy, infancy, randomized controlled trials, childhood, body composition, adiposity

## Abstract

Background: Vitamin D deficiency during pregnancy or infancy is associated with adverse growth in children. No systematic review has been conducted to summarize available evidence on the effect of vitamin D supplementation in pregnancy and infancy on growth and body composition in children. Objective: We aim to summarize the available evidence on the effect of vitamin D supplementation in pregnancy and infancy on child growth and body composition. Method: A systematic review and meta-analysis were performed on the effects of vitamin D supplementation during early life on children’s growth and body composition (bone, lean and fat). A literature search of randomized controlled trials (RCTs) was conducted to identify relevant studies on the effects of vitamin D supplementation during pregnancy and infancy on children’s body composition (bone, lean and fat) in PubMed, EMBASE and Cochrane Library from inception to 31 December 2020. A Cochrane Risk Assessment Tool was used for quality assessment. The comparison was vitamin D supplementation vs. placebo or standard care. Random-effects and fixed-effect meta-analyses were conducted. The effects are presented as mean differences (MDs) or risk ratios (RRs) with 95% confidence intervals (CIs). Results: A total of 3960 participants from eleven randomized controlled trials were eligible for inclusion. Vitamin D supplementation during pregnancy was associated with higher triceps skinfold thickness (mm) (MD 0.33, 95% CI, 0.12, 0.54; I^2^ = 34%) in neonates. Vitamin D supplementation during pregnancy or infancy was associated with significantly increased length for age z-score in infants at 1 year of age (MD 0.29, 95% CI, 0.03, 0.54; I^2^ = 0%), and was associated with lower body mass index (BMI) (kg/m^2^) (MD −0.19, 95% CI −0.34, −0.04; I^2^ = 0%) and body mass index z-score (BMIZ) (MD −0.12, 95% CI −0.21, −0.04; I^2^ = 0%) in offspring at 3–6 years of age. Vitamin D supplementation during early life was not observed to be associated with children’s bone, lean or fat mass. Conclusion: Vitamin D supplementation during pregnancy or infancy may be associated with reduced adiposity in childhood. Further large clinical trials of the effects of vitamin D supplementation on childhood body composition are warranted.

## 1. Introduction

There is growing interest regarding the association between early life vitamin D status with children’s growth, bone health, adiposity and muscle development. It is widely accepted that vitamin D plays a critical role in bone health by maintaining calcium homeostasis [1]. This function becomes especially important during pregnancy when the developing fetus is entirely dependent on the mother for accretion of roughly 30 g of calcium for skeletal purposes [2,3]. In addition to its calcium metabolic functions, mixed evidence suggests that infant adiposity and lean mass are in part determined by vitamin D status [2]. Vitamin D may also play a role in maintaining normal glucose homeostasis during pregnancy, thus preventing fetal macrosomia and excess deposition of subcutaneous fat [4]. Vitamin D receptors have been isolated in skeletal muscle tissues [5], and low vitamin D concentration is associated with proximal myopathy and reduced physical performance [6].

Several observational studies [7,8,9,10,11,12,13,14,15] on maternal vitamin D status and growth or body composition in offspring have been conducted. Low vitamin D concentrations were associated with lower birthweight [11]. Offspring exposed to higher maternal serum 25(OH)D concentrations had lower fat mass and higher bone mass during infancy [6]. In its most severe form, infants born to mothers who had vitamin D deficiency were at elevated risk of rickets. [16,17,18] While there are few observational studies relating postnatal muscle development to intrauterine 25(OH)D exposure, no association was reported between the two in adulthood in one study [19]. Another observational study concluded that prenatal vitamin D exposure may have a greater effect on muscle strength than on muscle mass in the development of offspring [6].

Considering the high prevalence of low vitamin D status during pregnancy and infancy [20,21,22,23], and the inconsistent results of the clinical trials [2,6], this systematic review and meta-analysis aimed to assess the effect of vitamin D supplementation in early life (pregnancy, lactation and infancy) on child growth, bone health, lean mass and adiposity.

## 2. Methods

We followed the guidelines for Preferred Reporting Items for Systematic Reviews and Meta-Analyses (PRISMA) [24].

### 2.1. Search Strategy

An electronic literature search of published studies was performed on PubMed, EMBASE and Cochrane Library up to 31 December 2020. The systematic literature search was based on the following search strategy: controlled vocabulary (i.e., MeSH Terms: “Vitamin D” [MeSH], “body composition” [MeSH]) as well as specific text words (including “vitamin D”, “calciferol”, “supplementation”, “pregnancy”, “infancy”, “growth”, “body composition”, “bone”, “lean mass”, “fat mass”) were included and systematically combined (AND/OR) with English language abstracts available. The details of the search strategy are presented in Table S1. Only English language papers on human clinical trials were considered. The reference lists of relevant reviews and studies were screened for additional articles [3,25,26,27,28].

### 2.2. Study Selection

Selected studies had to fulfill the following criteria to qualify for inclusion: (a) the study design is a randomized controlled trial (RCT) of vitamin D supplementation (800–5000 IU/day vs. placebo or standard care) or, in cases of co-intervention, with consistent additional supplements across treatment groups; (b) the study population are children; (c) the outcomes measured at least one of the following: bone mineral content (BMC), fat mass, lean mass, skinfold thickness, body mass index (BMI), body mass index z-score (BMIZ), weight for age z-score (WAZ), length for age z-score (LAZ) and head circumference for age z-score (HCAZ); (d) the study met the methodological quality assessment criteria for RCTs [29]. Studies were excluded if: (a) the outcome data were incomplete or impossible to compare with other studies; (b) there was no appropriate control group.

Two authors (K. M. and W. G. B.) independently searched for and assessed the eligibility of the electronic literature by initially screening titles and abstracts. Full-length articles of potential studies to be included were then obtained and read to make final inclusion or exclusion decisions. In case of disagreement, a third reviewer (S. Q. W.) was consulted.

### 2.3. Quality Assessment

Using the Cochrane Risk Assessment Tool, we evaluated the methodological quality of each included clinical trial based on the following criteria: random sequence generation, allocation concealment, blinding of participants and personnel, blinding of outcome assessment, incomplete outcome data, selective reporting and other biases [29]. We assigned each of the abovementioned items as having either a low, high or unclear risk of bias for each eligible RCT.

### 2.4. Data Extraction and Synthesis

A data extraction form was used to collect the information of the individual clinical trial regarding the study characteristics: the first author’s last name, year of publication, country of origin, study design, total sample size, characteristics of participants, initiation of supplementation, interventions and outcomes. Data were extracted by two reviewers independently following a per-protocol analysis.

All statistical analyses were performed using Review Manager (version 5.3). For interventional studies with multiple experimental groups receiving varying amounts of vitamin D supplements, data were merged to form only one experimental group per study. All outcomes in this analysis had continuous data. The mean, standard deviation and number of participants for both the control and experimental group of each studied outcome were used to calculate the sample size weighted mean difference (MD). The point estimate was illustrated by forest plots for each study with a 95% confidence interval (CI). Heterogeneity was assessed by calculating the I squared (I^2^) statistic. Results were merged using a fixed effects model for I^2^ less than 50%, and a random effects model was applied when I^2^ reached 50% or more. A *p*-value of less than 0.05 was considered significant for our systematic review.

## 3. Results

### 3.1. Study Selection

**Figure 1:**
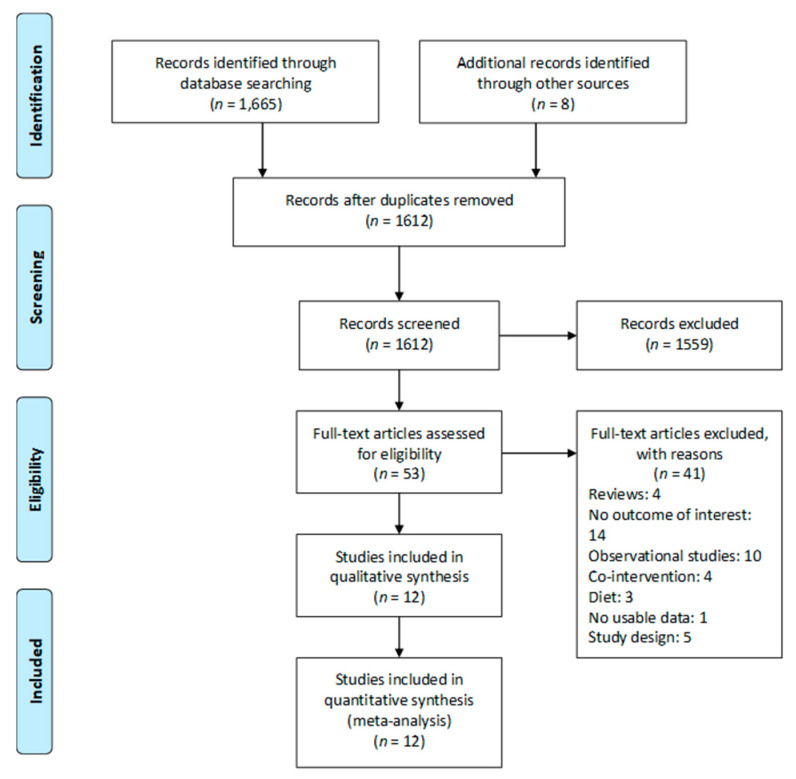
Preferred Reporting Items for Systematic Reviews and Meta-analyses (PRISMA) diagram.

Our search strategy identified 1665 potential publications. After screening the titles and abstracts, we read 53 full articles, of which 12 studies were included in this systematic review [3,26,27,30,31,32,33,34,35,36,37,38]. The selection process of the relevant literature is summarized in the Preferred Reporting Items for Systematic Reviews and Meta-Analyses (PRISMA) Flow Diagram (Figure 1).

### 3.2. Characteristics of Included Trials

Twelve RCTs [3,26,27,30,31,32,33,34,35,36,37,38,39,40] involving a total of 4583 participants were included in this systematic review. Nine trials conducted vitamin D supplementation during pregnancy [3,26,27,30,31,33,34,36,40], and three trials performed vitamin D supplementation during infancy [30,34,38]. One study involved a subgroup of vitamin D supplementation in lactation [37]. One study [32] followed up children at 1 (Hazell 2014) [33] and 3 (Hazell 2017) [34] years of age. Six studies [3,26,36,37,38,39] were placebo-controlled; four [27,30,34,40] were comparisons between higher vs. lower doses of vitamin D, and the lowest-dose group (400 IU/day) (this low dose is part of the standard care) served as the control; and two [33,36] involved control groups without supplements. All intervention groups were supplemented with cholecalciferol, except two studies [31,39] that used ergocalciferol supplementation. One study conducted vitamin D and calcium supplementation in all treatment groups, but the doses of vitamin D were different between intervention groups and the control group (intervention: 60,000 IU/4 weeks or 60000 IU/8 weeks; Control: 400 IU/day) [27]. Three of the RCTs [32,34,35] were follow-up studies. Details of the characteristics of included studies are shown in Table 1.

### 3.3. Risk of Bias of Included Clinical Trials

Risk of bias of included clinical trials is presented in Table S2. Participation completion rates were especially low in the two RCT follow-up studies in infants at 43.9% [38] and 66% [33], respectively. For random sequence generation, there were eight studies with low risk of bias and two studies in pregnancy [35,39] with unclear risk of bias. For allocation concealment, there were nine studies with low risk of bias, one study with high risk of bias [31] and one study [35] with unclear risk of bias. For blinding of participants and personnel, there were ten studies with low risk of bias and one study [33] with unclear risk of bias. For blinding of outcome assessment, there were eight studies with low risk of bias and three studies with unclear risk of bias. For incomplete outcome data, there were eight studies with low risk of bias, and three studies [with high risk of bias. For selective outcome data, all studies had a low risk of bias except for Brooke et al. [39], which had a high risk of bias. For other sources of bias, there was one study [31] with a high risk of bias; the rest had a low risk of bias.

### 3.4. Bone Mineral Content (BMC)

**Figure 2:**
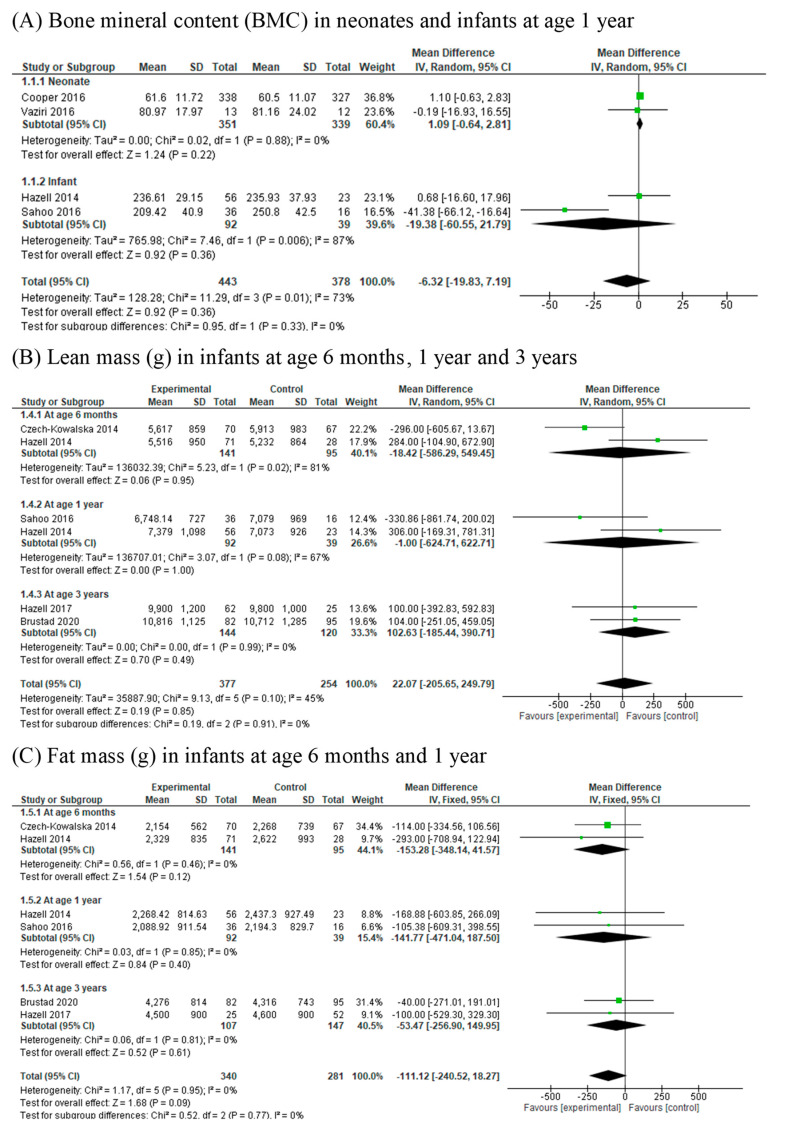
Forest plots of summary crude risk ratios of the association between vitamin D supplementation in early life and childhood body composition ((**A**) Bone mineral content; (**B**) Lean mass; (**C**) Fat mass).

Whole-body BMC (g) was examined in five RCTs [3,26,27,31,33] involving 1444 and 1349 mother–newborn pairs. Dual-energy X-ray absorptiometry (DXA) was used in all four studies to assess bone parameters. Bone health assessment was performed in the infants at one week, three weeks, between 12 and 16 months, and 3–6 years. There was no association between vitamin D supplementation during pregnancy with whole-body BMC (gram) in neonates (MD 1.09, 95% CI 0.64, 2.81; I^2^ = 0) and BMC (gram) in infants at 1 year of age (MD −19.38, 95% CI −60.55, 21.79; I^2^ = 73%) (Figure 2A).

### 3.5. Lean Mass (g) and Lean Mass Percentage (%)

Lean mass (gram) was reported in six RCTs [27,30,31,33,34,40] involving 631 participants. Lean mass was measured using DXA at approximately seven days, 6 months, between 12 and 16 months and at 36 months. Vitamin D supplementation was not associated with total lean mass in infants at ages 6 months (MD, −18.42, 95% CI −586.29, 549.45; I^2^ = 81%), 1 year (MD −1.00, 95% CI −624.71, 622.71, I^2^ = 67%) and 3 years (MD 102.63, 95% CI −185.44, 390.71, I^2^ = 0%) (Figure 2B). The data could not be pooled when lean mass was not assessed at similar ages. Cooper et al. [26] observed that the lean mass in the infants born to mothers assigned to cholecalciferol supplementation were not significantly different from mothers in the placebo group. One study [33] showed that lean mass percentage in infants did not differ with vitamin D supplementation.

### 3.6. Fat Mass (g) and Fat Mass Percentage (%)

Fat mass (g) was reported in five RCTs [27,30,34,35,40] involving 621 participants. Fat mass was measured using DXA at approximately seven days, 6 months, 12–16 months and 3 years of age. Vitamin D supplementation was not associated with total body fat mass (g) in the infants at ages 6 months (MD, −153.28, 95% CI −348.14 to 41.57, I^2^ = 0%), 1 year (MD, −141.77; 95% CI, −471.04 to 187.50, I^2^ = 0%) and 3 years (MD, −53.47; 95% CI, −256.90 to 149.95, I^2^ = 0%) (Figure 2C). The data could not be pooled when fat mass was not assessed at similar ages. Three RCTs involving 360 participants reported the outcome of fat mass percentage (%) at ages 1 year and 3–6 years. Vitamin D supplementation was not associated with fat mass percentage (%) in the infants at 1 year of age (MD −0.92, 95% CI −3.65, 1.81, I^2^ = 0).

### 3.7. Skinfold Thickness

**Figure 3:**
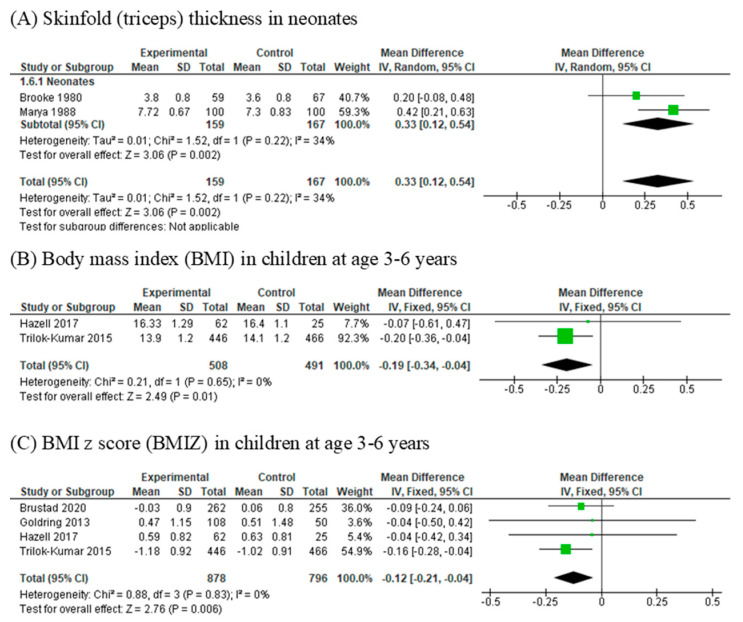
Forest plots of summary crude risk ratios of the association between vitamin D supplementation in early life and adiposity indicators ((**A**) Skinfold thickness, mm; (**B**) Body mass index; (**C**) Body mass index z score) in children.

Skinfold (triceps) thickness (mm) was assessed in three RCTs [35,38,39] with 555 participants. Outcomes were measured at birth in two RCTs [35,39] and between the age of three and six years in the third study [38]. Meta-analysis could only be performed for the two RCTs that measured outcomes at birth due to age disparity for outcome measurements with the third study. Neonates whose mothers had been supplemented with vitamin D had significantly higher skinfold thickness (mm) than those who had not (MD 0.33, 95% CI 0.12, 0.54). There was no significant heterogeneity (I^2^ = 34%) (Figure 3A). Trilok-Kumar et al. [38] reported no association between infancy supplementation of vitamin D and skinfold thicknesses.

### 3.8. Body Mass Index (BMI)

Two RCTs [34,38] involving 999 participants reported the outcome of BMI (kg/m^2^). Vitamin D supplementation (vs. placebo or standard care) in infancy was associated with significantly lower BMI (kg/m^2^) between the ages of 3 and 6 years (MD −0.19, 95%CI −0.34, −0.04). Heterogeneity was not significant (I^2^ = 0%) (Figure 3B).

### 3.9. Body Mass Index Z-Score (BMIZ)

Four RCTs [31,34,38,40] involving 1674 participants reported the outcome of infant BMIZ. Offspring who had prenatal or postnatal vitamin D supplementation (vs. placebo or standard care) had a significantly lower BMIZ at three to six years old (MD −0.12; 95% CI −0.21, −0.04). No significant heterogeneity was detected (I^2^ = 0%) (Figure 3C).

### 3.10. Weight for Age Z-Score (WAZ) and Length for Age Z-Score (LAZ)

WAZ was examined in six RCTs [27,31,32,34,35,37], and LAZ was examined in four RCTs [23,28,30,39] involving 2495 and 1196 participants, respectively. Both outcomes were assessed in children at ages one year [34], between 12 and 16 months [27], three years [32] and between three and six years [31,35]. Due to age differences, results were separately merged for outcomes examined at ages 12–18 months [27,34] and three to six years [32,35]. There was no significantly difference between the intervention group in the outcome WAZ in children at ages 1 year (MD −0.07; 95%CI −0.20 to 0.07) and 3–6 years (MD −0.06, 95% CI −0.18, 0.06). LAZ was higher in infants 1 year of age in the vitamin D supplementation group compared with the control group (MD 0.29, 95% CI 0.03, 0.54; I^2^ = 0%); however, there was no significant difference in LAZ in children at 3–6 years between the two groups (MD 0.04, 95%CI −0.08, 0.16; I^2^ = 0%).

### 3.11. Head Circumference for Age Z-Score (HCAZ)

HCAZ was measured in two RCTs [27,34] with 183 infants. No association was found between maternal vitamin D supplementation and HCAZ (MD 0.12, 95%CI −0.18, 0.42). There was no significant heterogeneity.

## 4. Discussion

### 4.1. Statement of Main Findings

This is the first systematic review and meta-analysis of the effects of vitamin D supplementation during early life (during pregnancy, lactation or infancy) on children’s body composition (bone health, lean mass and adiposity). We found that vitamin D supplementation during pregnancy was associated with higher skinfold thickness in neonates. Vitamin D supplementation in early life was associated with significantly higher length for age z-score in infants at 1 year of age, and was associated with lower BMI and BMI z-score in offspring at 3 to 6 years of age. From current evidence, vitamin D supplementation during early life was not found to be associated with children’s BMC, lean mass (g, %), WAZ and HCAZ. We found that vitamin D supplementation during early life had a consistent trend to decrease body fat mass (g, %), although the 95% CI confidence intervals included the null effect. There was no heterogeneity (I^2^ = 0) across studies. These null effects may be due to the small sample size in the included trials. Large well-designed clinical trials are needed to confirm the above associations.

### 4.2. Importance and Implications

This systematic review added to the existing literature by including a greater number of recent RCTs and the first systematic review and meta-analysis of RCTs on the effects of vitamin D supplementation during early life (during pregnancy, lactation or infancy) on the outcomes of children’s bone (whole-body BMC), muscle (lean mass and lean mass percentage), adiposity (skinfold thickness, fat mass and fat mass percentage) and growth (age and sex specific indicators: BMIZ, WAZ, LAZ and HCAZ). The results show that vitamin D supplementation in early life was associated with higher skinfold thickness in neonates, higher LAZ in infants and lower BMIZ in children at 3–6 years of age, suggesting that vitamin D in early life may play an important role in children’s adiposity development, which may have a public health implication for the early intervention or prevention of childhood overweight/obesity and related cardiometabolic health issues.

### 4.3. Comparison with Previous Studies

There are several systematic reviews [1,11,25,28,41,42,43,44,45] on the effects of maternal vitamin D supplementation intake or status during pregnancy on maternal, neonatal or infant health outcomes. In contrast, we could not identify any meta-analysis examining the effects of vitamin D supplementation during early life on child body composition. One narrative review [46] described the relationship between vitamin D and BMD and found that it is inconsistent across studies; however, the authors did not perform a meta-analysis. While most studies [1,25,28,41,44,45] included anthropometric measures, such as birthweight, birth length and head circumference, none of them reported the respective sex-specific and age-specific z-scores.

Harvey et al. published a comprehensive review on both observational and clinical trials of the role of vitamin D during pregnancy in perinatal outcomes (such as birthweight, birth length, head circumference, anthropometry and body composition and low birthweight) [25]. Like our review, their study [25] showed that child BMC was not affected by supplementation of vitamin D, and the results were inconsistent regarding skinfold thickness. However, our systematic review included more recent studies, and the meta-analysis was based only on RCTs. Another review by Curtis et al. evaluated the link between prenatal vitamin D supplementation and child bone development, but lacked results on other body composition outcomes, such as fat and lean mass [2]. This study found that achieving a higher level of serum 25 hydroxyvitamin D [25(OH)D] in pregnancy might have beneficial effects on the bone development of offspring. However, there are not enough high quality RCTs to assess this, and the timing of assessment is variable among existing trials. No association of vitamin D with BMC in early childhood could preclude an effect on adolescence and adulthood. Longer-term follow-up is needed.

Our previous meta-analysis showed that maternal low vitamin D status during pregnancy was associated with lower birthweight, and higher weight at 9 months of age, which indicates that prenatal vitamin D status was related with accelerated weight gain during infancy that may be linked to increased adiposity in offspring [11]. Our other systematic review demonstrates that vitamin D supplementation during pregnancy increased birthweight and reduced the risk of small for gestational age [43]. However, the above two studies did not study the effect of vitamin D on bone health, lean mass and fat mass. Despite four RCTs [31,32,35,36] in this current review showing that BMI and BMIZ were lower in participants who received prenatal or postnatal vitamin D supplementation, it is important to note that children were studied around the usual age of BMI and adiposity rebound, which occur at approximately 4 and 6 years of age, respectively [35,47]. The long-term effects of vitamin D supplementation during early life on BMI and BMIZ are unclear. More high quality RCTs are required to assess the link between vitamin D supplementation and lean mass in early life.

### 4.4. Mechanisms

Vitamin D is important for the differentiation of mesenchymal stem cells into adipocytes. Early life vitamin D adequacy promotes the conversion of preadipocyte maturation to form myocytes rather than mature adipocytes. A study performed on mice showed that offspring gestated in a vitamin D-deficient diet possess larger visceral body fat pads and greater susceptibility to high fat diet-induced adipocyte hypertrophy [48]. Moreover, greater nuclear receptor peroxisome proliferator-activated receptor gamma (Pparg) expression in visceral adipose tissue was also observed in this study performed on mice. The nuclear receptor PPARG takes part in both adipogenesis and lipid storage [49,50,51].

While direct supplementation of vitamin D did not lead to a difference in lean mass between control and experimental groups in this meta-analysis, Hazell et al. showed that higher vitamin D status correlates with a leaner body composition; infants with a plasma 25(OH)D3 concentration above 75 nmol/L did not differ in lean mass and fat mass compared with those below 75 nmol/L [37]. Previous work has shown that the biologically active form of vitamin D, 1,25(OH)-2D, binds to vitamin D receptors to signal gene transcription and sensitize the Akt/mTOR pathway involved in protein synthesis [32,52].

### 4.5. Strengths and Limitations

Our systematic review has its strengths. It is the first systematic review and meta-analysis of randomized controlled trials to assess the effectiveness of vitamin D supplementation during early life (pregnancy and/or infancy) on body composition. Risk of bias in the RCTs was evaluated to ensure quality of the included studies. This study has some limitations. First, we included eleven RCTs of vitamin D supplementation in early life on children’s body composition; the outcome measures were quite different across individual studies, and therefore, for each outcome, there were only a few RCTs. Second, outcome assessment was performed in children at different ages, which made pooling the data impossible for certain outcomes. The baseline vitamin D status, timing and the dose of vitamin D supplementation administered during pregnancy or infancy also differed across studies. There was a lack of data on visceral vs. subcutaneous adiposity. Moreover, most trials had no information on the compliance with vitamin D supplementation. Finally, small sample sizes and the loss to follow-up were additional limiting factors.

## 5. Conclusions

This systematic review of randomised clinical trials suggests that that vitamin D supplementation during pregnancy is associated with higher skinfold thickness in neonates. Vitamin D supplementation during pregnancy or infancy is associated with lower BMI and BMI z-score in offspring at 3 to 6 years of age. Based on current published clinical trials, vitamin D supplementation in early life is not observed to be associated with bone, lean and fat mass by DXA. Future large well-designed double blinded RCTs are needed to assess the effectiveness of vitamin D supplementation in early life on children’s bone health, lean mass and adiposity.

## Figures and Tables

**Table 1:** Characteristics of the included studies ^1^.

Study	Country	Study Design	Total Sample Size (*n*)	Participants	Baseline 25(OH)D (nmol/L)	Initiation and Duration of Supplementation	Interventions	Compliance (% of Dosages Taken)	Outcomes
Brooke 1980 [39]	United Kingdom	RCT	126	Pregnant Asian women	NA	Third trimester	Ergocalciferol, 1000 IU/day vs. placebo	NA	Neonatal anthropometry (triceps skinfold thickness)
Brustad 2020 [40]	Denmark	RCT	517	Pregnant women	77.5 nmol/L	24 weeks of gestation to 1 week postpartum	Cholecalciferol 2800 IU/day vs. 400 IU/day	74%	BMC, fat and lean mass, and BMIZ in children at 3 years of age
Cooper 2016 [26]	United Kingdom	RCT	965	Pregnant women gestation age less than 17 weeks, serum 25(OH)D at 25–100 nmol/L at 10–17 weeks’ gestation	Mean: 45.8 nmol/L <50 nmol/L:42.2%	14 weeks of gestation or before 17 weeks of gestation until delivery	Cholecalciferol, 1000 IU/day vs. placebo	70%	Neonatal whole-body BMC, fat mass and lean mass by DXA
Czech-Kowalska 2014 [30]	Poland	RCT	137	Healthy women who delivered at term, a single neonate and breastfed for the next 6 months	Mean: 37.9 nmol/L <50 nmol/L:68%	At delivery for 6 months	Cholecalciferol 1200 IU/day vs. 400 IU/day	82%	Fat mass and lean body mass by DXA in infants at 6 months
Goldring 2013 [31]	United Kingdom	RCT	158	Women presenting at 27 weeks gestation	<25 nmol/L:44.9%	27 weeks of gestation until delivery or single dose	Ergocalciferol 800 IU/day or cholecalciferol single oral dose of 200,000 IU vs. no treatment (control)	NA	BMIZ in children at 3 years of age
Gallo 2013 [32] Hazell 2014 [33] Hazell 2017 [34]	Canada	RCT	132	1 month old healthy, breastfed infants	Mean: 62.1 nmol/L	1 month old to 12-months (for 11 months)	Cholecalciferol of 800, 1200 or 1600 IU/day vs. 400 IU/day	84%	Anthropometry (WAZ, HAZ), BMI, BMIZ, body composition (BMC, lean mass and fat mass) by DXA at 1 and 3 years of age
Marya 1988 [35]	India	RCT	200	Pregnant women aged 22–35 years without complications	NA	Administration of 2 doses, one at the 7th and the other at the 8th month of gestation	Cholecalciferol 600,000 IU/dose vs. unsupplemented control	NA	Neonatal anthropometry (triceps skinfold)
Roth 2013 [36]	Bangladesh	RCT	134	Pregnant women at 26 to 29 weeks gestation	Mean: 41.1 nmol/L	Third trimester	Cholecalciferol 35,000 IU/week vs. placebo	NA	Anthropometry (WAZ, LAZ, HCAZ) at 1 year of age
Roth 2018 [37]	Bangladesh	RCT	1164	Pregnant women and their infants	Mean: 27.5 nmol/L	Supplementation of pregnant women from 17 to 24 weeks of gestation until birth and, in one subgroup, 26 weeks of postnatal supplementation in infants	Cholecalciferol 4200 IU/week vs. 16,800 IU/week vs. 28,800 IU/week vs. placebo	At least 90%	Anthropometry (WAZ, LAZ, HCAZ, BMIZ) at birth and at 1 year of age
Sahoo 2016 [27]	India	RCT	52	Healthy pregnant women less than 20 weeks of gestation	Mean: 28.2 nmol/L <50 nmol/L: 87%	14–20 weeks of gestation until delivery	Cholecalciferol 60,000 IU/4 weeks or 60,000 IU/8 weeks vs. 400 IU/day	NA	Anthropometry (WAZ, LAZ, HCAZ), whole body BMC, lean mass and fat mass by DXA at 1 year of age
Trilok-Kumar 2015 [38]	India	RCT	912	Healthy neonates	Mean: 36 nmol/L <50 nmol/L: 73%	Infants at 7 days of age for 6 months	Cholecalciferol 1500 IU/week vs. placebo	NA	Anthropometry (WAZ, HAZ, triceps skinfold), BMI, BMIZ, bone structure and strength, deuterium dilution test of body composition on a subset (*n* = 229) at age 3–6 years
Vaziri 2016 [3]	Iran	RCT	25	Healthy pregnant women	Mean: 30.2 nmol/L	26–28 weeks of gestation until childbirth	Cholecalciferol 2000 IU/day vs. placebo	NA	Whole-body: BMC by DXA in offspring at birth, 4th and 8th weeks of age

^1^ 25(OH)D, 25 hydroxivitamin D; BMC, bone mineral content; DXA, dual-energy X-ray absorptiometry; BMI, body mass index; BMIZ, body mass index z-score; HCAZ, head circumference for age z-score; LAZ, length for age z-score; NRS, non-randomized controlled study; RCT, randomized controlled trial; WAZ, weight for age z-score.

## Supplementary Materials

The following are available online at https://www.mdpi.com/2072-6643/13/2/524/s1, Table S1: Search strategy, Table S2: Assessment of Bias Risk of Randomized Controlled Trials.
