# Supplementary material for: Effect of Vitamin D Supplementation in Early Life on Children’s Growth and Body Composition: A Systematic Review and Meta-Analysis of Randomized Controlled Trials
Source: Nutrients. 2021 Feb 5;13(2):524. doi: 10.3390/nu13020524 (PMC7914476; doi:10.3390/nu13020524)
Supplement: Supplementary file 1 [file nutrients-13-00524-s001.pdf]

Table S1. Search strategy

#	Search	Details
1	Vitamin D	"Vitamin D"[Mesh] OR vitamin D*[Text Word] OR "25-hydroxyvitamin D"[Substance] OR "25-hydroxyvitamin D"[Text Word])
2	Pregnancy	"Pregnancy"[Mesh] OR "PREGNANT" [Text Word] OR "PREGNANCY" [Text Word] OR "ANTEPARTUM" [Text Word] OR "PRENATAL" [Text Word] OR "ANTE-PARTUM" [Text Word] OR "PRE-NATAL" [Text Word] OR "PREPART" [Text Word] OR "ANTENATAL" [Text Word]
3	Lactation	"Lactation"[Mesh] OR "Breastfeeding"[Mesh] OR "Breastfeeding" [Text Word] OR "breast feeding" [Text Word] OR "human milk" [Text Word] OR "breast milk" [Text Word] OR "POSTPARTUM" [Text Word] OR "POSTNATAL" [Text Word] OR "POST-PARTUM" [Text Word] OR "POST-NATAL" [Text Word]
4	Infancy	"Infancy"[Mesh] OR "Infant" [Text Word] OR "PREGNANCY" [Text Word] OR "ANTEPARTUM" [Text Word] OR "PRENATAL" [Text Word] OR "ANTE-PARTUM" [Text Word] OR "PRE-NATAL" [Text Word] OR "PREPART" [Text Word] OR "ANTENATAL" [Text Word]
5	Neonatal and child growth	"Growth"[Mesh] OR "Growth Disorders"[Mesh] OR "Nutritional Status"[Mesh] OR stunt[Text Word] OR underweight[Text Word] OR wast[Text Word] OR "Body Height"[Mesh] OR "Body Weight"[Mesh] OR "height"[Text Word] OR "weight"[Text Word] OR "Anthropometry"[Mesh] OR Anthropom*[Text Word]
6	Body composition	" Body composition "[Mesh] OR "muscle, skeletal"[Mesh] OR OR "muscle mass"[Text Word] OR "lean mass"[Text Word] OR "fat mass"[Text Word] OR "adiposity"[Text Word] OR " body mass index"[Text Word] OR "BMI"[Text Word] OR "skinfold thickness"[Text Word] OR "skinfold measurement"[Text Word]

The search was restricted to human studies in English language. For the search we included #1 AND #2-4 AND #5-6.

Table S2. Assessment of Bias Risk of Randomized Controlled Trials

Study	Sequence generation	Allocation concealment	Blinding of participants and personnel	Blinding of outcome assessment	Incomplete outcome data	Selective outcome data	Other sources of bias
Brooke 1980[30]	Unclear	Low	Low	Low	Low	High	High
Brustad 2020[40]	Low	Low	Low	Low	Low	Low	Low
Cooper 2016[26]	Low	Low	Low	Low	Low	Low	Low
Czech-Kowalska 2014[31]	Low	Low	Low	Low	Low	Low	Low
Goldring 2013[32]	Low	High	Low	Low	Low	Low	High
Hazell 2014[34]	Low	Low	Low	Unclear	High	Low	Low
Marya 1988[36]	Unclear	Unclear	Unclear	Unclear	Low	Low	Low
Roth 2013[37]	Low	Low	Low	Low	Low	Low	Low
Roth 2018[38]	Low	Low	Low	Low	Low	Low	Low
Sahoo 2017[27]	Low	Low	Low	Low	High	Low	Low
Trilok-Kumar 2015[39]	Low	Low	Low	Unclear	High	Low	Low
Vaziri 2016[3]	Low	Low	Low	Low	Low	Low	Low
